# CXCR5 down-regulation alleviates cognitive dysfunction in a mouse model of sepsis-associated encephalopathy: potential role of microglial autophagy and the p38MAPK/NF-κB/STAT3 signaling pathway

**DOI:** 10.1186/s12974-021-02300-1

**Published:** 2021-10-28

**Authors:** Yanan Shen, Yuan Zhang, Jiayue Du, Baochun Jiang, Tao Shan, Haojia Li, Hongguang Bao, Yanna Si

**Affiliations:** 1 Department of Anesthesiology, Nanjing First Hospital, Nanjing Medical University, Nanjing, 210006 Jiangsu China; 2 Institute of Pain Medicine and Special Environmental Medicine, Nantong University, Nantong, 226019 Jiangsu China

**Keywords:** Sepsis-associated encephalopathy, Neuroinflammation, Autophagy, CXCR5, p38MAPK

## Abstract

**Background:**

Cognitive deficits are common in patients with sepsis. Previous studies in sepsis-associated encephalopathy (SAE) implicated the C-X-C chemokine receptor type (CXCR) 5. The present study used a mouse model of SAE to examine whether CXCR5 down-regulation could attenuate cognitive deficits.

**Methods:**

Sepsis was induced in adult male C57BL/6 J and *CXCR5*^*−/−*^ mice by cecal ligation and puncture (CLP). At 14–18 days after surgery, animals were tested in a Morris water maze, followed by a fear conditioning test. Transmission electron microscopy of hippocampal sections was used to assess levels of autophagy. Primary microglial cultures challenged with lipopolysaccharide (LPS) were used to examine the effects of short interfering RNA targeting CXCR5, and to investigate the possible involvement of the p38MAPK/NF-κB/STAT3 signaling pathway.

**Results:**

CLP impaired learning and memory and up-regulated CXCR5 in hippocampal microglia. CLP activated hippocampal autophagy, as reflected by increases in numbers of autophagic vacuoles, conversion of microtubule-associated protein 1 light chain 3 (LC3) from form I to form II, accumulation of beclin-1 and autophagy-related gene-5, and a decrease in p62 expression. CLP also shifted microglial polarization to the M1 phenotype, and increased levels of IL-1β, IL-6 and phosphorylated p38MAPK. CXCR5 knockout further enhanced autophagy but partially reversed all the other CLP-induced effects, including cognitive deficits. Similar effects on autophagy and cytokine expression were observed after knocking down CXCR5 in LPS-challenged primary microglial cultures; this knockdown also partially reversed LPS-induced up-regulation of phosphorylated NF-κB and STAT3. The p38MAPK agonist P79350 partially reversed the effects of CXCR5 knockdown in microglial cultures.

**Conclusions:**

CXCR5 may act via p38MAPK/NF-κB/STAT3 signaling to inhibit hippocampal autophagy during sepsis and thereby contribute to cognitive dysfunction. Down-regulating CXCR5 can restore autophagy and mitigate the proinflammatory microenvironment in the hippocampus.

## Background

Sepsis is a complex, life-threatening syndrome involving multiple organ systems, and more than half of sepsis patients are admitted to an intensive care unit, accounting for 20–30% of all hospital deaths [1–3]. Sepsis-associated encephalopathy (SAE) occurs in approximately 70% of sepsis patients [4], and is associated with increased morbidity and mortality [5, 6]. Clinical manifestations include prolonged cognitive impairment as well as psychological disorders (e.g., anxiety and depression) [6].

Mechanisms for the development SAE are not fully understood. A leading hypothesis is that sepsis induces neuroinflammation in the central nervous system (CNS), leading to brain damage and dysfunction [7, 8]. Microglia, the major resident immune cells in the CNS are activated upon sepsis, and shift from a “surveillance” phenotype to a proinflammatory M1 phenotype to release inflammatory signals [9, 10]. In this way, microglial activation exacerbates neuronal injury and impairs learning and memory [11]. In mouse models, SAE could be alleviated by blocking microglial activation via inhibiting the IL-17A/IL-17R inflammatory pathway [12], injecting attractylone to polarize microglia toward the M2 phenotype [13], or injecting minocycline [14] or the ginsenoside Rg1 [15] to inhibit neuroinflammation.

Autophagy, an evolutionarily conserved catabolic process to recycle damaged or senescent organelles and proteins [16], is elevated upon sepsis in hepatocytes [17], cardiomyocytes [18], as well as the CNS [10, 19]. The process of autophagy includes lysosome activation, autophagosome formation, conversion of microtubule-associated protein 1 light chain 3 (LC3) from form I to form II, and a reduction in levels of Beclin-1, LAMP-1 and Rab7 [19]. Deletion of the genes that encode autophagy-related proteins, such as autophagy-related gene-5 (Atg-5), exacerbates the production of pro-inflammatory cytokines in multiple tissues following sepsis [20], suggesting increased autophagy is a compensatory response that limits sepsis-induced tissue damage.

Deficiency in the C-X-C motif chemokine receptor 5 (CXCR5) in retinal pigment epithelium cells has been linked to up-regulation of autophagy [21]. A previous study in a mouse model of sepsis from this laboratory showed that CXCR5 contributes to hippocampal neuroinflammation, subsequently leading to hippocampal neurogenesis disorder and cognitive impairment, and CXCR5 deficiency alleviates sepsis-induced deficits in hippocampal neurogenesis and cognitive function [22]. In the current study, we examined whether down-regulating CXCR5 could alleviate cognitive deficits induced by sepsis by regulating autophagy in hippocampus. Considering previous studies that linked CXCR5 to p38MAPK activation [23], and p38MAPK to autophagy and neuroinflammation [24], the mechanistic investigation in the current study focused on the p38MAPK/NF-κB/STAT3 pathway.

## Methods

### Animals

Study protocols involving animal subjects were approved by the Animal Ethics Committee of Nanjing Medical University (approval #: IACUC-2004043). All experiments were conducted in strict accordance with institutional guidelines. Adult male C57BL/6 J mice (6–8 weeks of age, 20–25 g) and neonatal pups (3–4 days of age, used in experiments involving primary culture) were obtained from Qinglongshan Animal Breeding Farm (Nanjing, China). *CXCR5*^*−/−*^ mice [B6.129S2 (Cg)-*CXCR5* ^tm1Lipp/J^, stock number 006659] with a B6 background were kindly provided by Professor Yongjing Gao (Institute of Pain Medicine, Nantong University, China) [23]. Mice were housed in a pathogen-free facility in the Experimental Animal Center at Nanjing First Hospital, and maintained under a 12-h light–dark cycle with ad libitum access to standard food and water.

### Study design

**Fig. 1:**
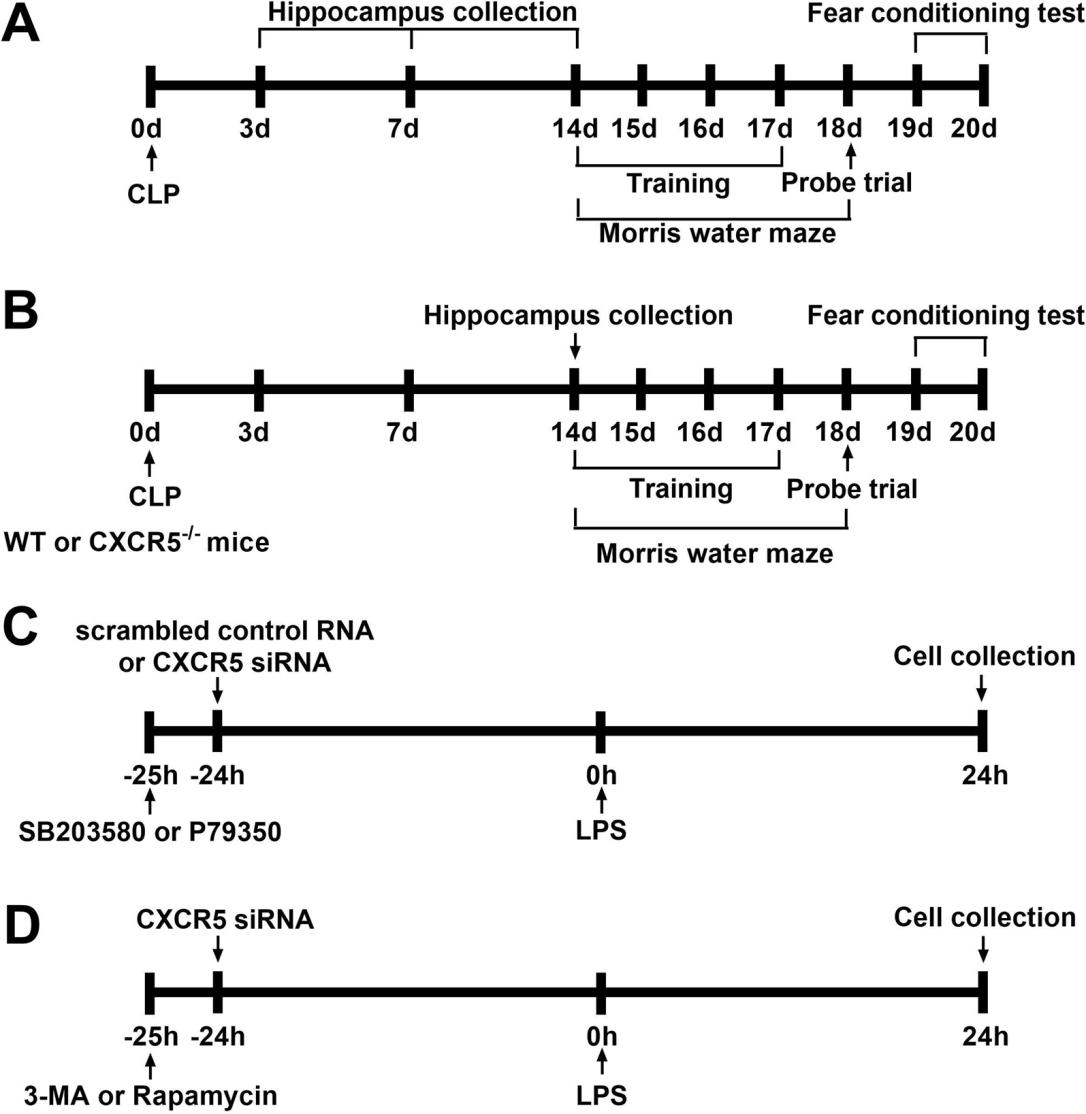
Schematic illustration of the experimental design. **a** Timeline of WT mice that underwent sham surgery or CLP. **b** Timeline of WT or *CXCR5*^*−/−*^ mice that underwent sham surgery or CLP. **c** Timeline of LPS-treated microglial cells treated with CXCR5 siRNA with or without pretreatment with the p38MAPK inhibitor SB203580 or agonist P79350. **d** Timeline of LPS-treated microglial cells treated with CXCR5 siRNA with or without pretreatment with the autophagy inhibitor 3-MA or agonist rapamycin. *CLP* cecal ligation and puncture, *CXCR5* C-X-C chemokine receptor type 5, *LPS* lipopolysaccharide, *siRNA* short interfering RNA, *3-MA* 3-methyladenine, *WT* wild-type

This study consisted of both in vivo and in vitro experiments. The in vivo experiments were conducted in mice (C57BL/6 J or *CXCR5*^*−/−*^) with cecal ligation and puncture (CLP; Fig. 1A, B). A group of C57BL/6 J mice undergoing sham surgery was included as a control. Tests for cognitive functions included a standard 5-day Morris water maze (14 days after CLP) and then a fear conditioning test (19 days after CLP). Separate groups of mice were killed 3, 7, and 14 days after CLP for Western blotting, transmission electron microscopy and immunohistochemistry analyses in the hippocampus. The in vitro experiments were conducted in primary microglial cells (Fig. 1C, D). Briefly, cells were seeded at a density of 3.5 × 10^5^ cells/mL in serum-free DMEM (Thermo Fisher Scientific, Waltham, MA, USA) into 6-well plates containing coverslips, incubated at 37 ℃ overnight, and then exposed to lipopolysaccharide (LPS; Thermo Fisher Scientific) at a concentration of 200 ng/mL for 24 h [13]. In some experiments, cultures were pretreated with short interfering RNA (siRNA) targeting CXCR5 (5′-CUGGACAGAUUGGACAACU-3′; GenePharma, Shanghai, China) or with scrambled sequence (5’-UUCUCCGAACGUGUCACGU-3′; GenePharma) at 24 h before LPS challenge [22]. CXCR5 knockdown was verified using Western blot analysis. The siRNAs (20 pmol) were dissolved in 100 μL serum-free OptiMEM, mixed with 1 μL Lipofectamine 2000 (Invitrogen, Carlsbad, CA, USA), and added to the cultures. In some experiments involving CXCR5 siRNA, cells were pretreated with the p38MAPK inhibitor SB203580 (10 mmol/L; Sigma-Aldrich, St Louis, MO, USA), the p38MAPK agonist P79350 (50 mmol/L; Calbiochem, La Jolla, CA, USA) or vehicle for 1 h prior to adding CXCR5 siRNA administration. In a separate set of experiments involving CXCR5 siRNA, cells were treated with the autophagy inhibitor 3-methyladenine (3-MA) (10 mmol/L; Monmouth Junction, NJ, USA) or agonist rapamycin (1 nmol/L; Sigma-Aldrich) at 25 h before LPS challenge to examine the role of autophagy in the effects of CXCR5 knockdown. Cells were collected at 24 h after LPS treatment for Western blotting and immunofluorescence analysis.

### CLP

CLP was conducted as previously described [25]. Briefly, mice were anesthetized with intraperitoneal injection of sodium pentobarbital (40 mg/kg). After disinfection, an incision 2–3 cm long was made at 1.5 cm below the xiphoid to expose the abdominal cavity. The cecum was isolated and ligated at half the distance between the distal pole and the base of the cecum, punctured with a 22-gauge needle, and gently squeezed to force the fecal contents into the peritoneal cavity. The cecum was returned to the peritoneal cavity, and the abdomen was closed using 3–0 silk sutures. In the case of sham-operated mice, the abdominal cavity was opened to expose the cecum without ligation or puncture.

Core body temperature was maintained at 37 ± 0.5 ℃ using a heating blanket during surgery. At the end of surgery, mice received 40 ml/kg sterile saline via subcutaneous injection and returned to home cage with a warm cotton pad and free access to food and water. No antibiotics were given.

### Primary microglial culture

Primary microglia cultures were prepared from neonatal mouse pups as described previously [26, 27]. Pups were disinfected with 70% alcohol, brains were harvested, and the cerebral cortex was carefully dissected and washed in ice-cold D-Hanks Balanced Salt Solution (Thermo Fisher Scientific) supplemented with 100 U/mL penicillin and 100 μg/mL streptomycin (Sigma-Aldrich). Tissue was dissociated by gentle trituration in Dulbecco’s modified Eagle’s medium (DMEM; Sigma-Aldrich) containing 10% heat-inactivated fetal bovine serum (Sigma-Aldrich) and antibiotics. The cell suspension was filtered through a 100-μm cell sieve (Becton Dickinson AB, Stockholm, Sweden) and centrifuged at 864 *g* for 5 min at 4 ℃. The pellet was suspended in DMEM, then incubated in a 75-mL culture flask (Thermo Fisher Scientific) for 14 days in a humidified incubator at 37 ℃ in an atmosphere containing 5% CO_2_. Next, the flask was shaken at 100 rpm at 37 ℃ for 6 h on a rotary shaker to harvesting microglia with centrifugation (288 *g* at 4 ℃ for 5 min).

### Morris water maze

Learning and memory were examined using a standard 5-day Morris water maze test, as described previously [28]. A circular water pool (125-cm diameter and 40-cm height) was filled with opaque water to a depth of 30 cm and maintained at 23 ± 1 ℃. The escape platform (10-cm diameter) was placed 1 cm below the water surface in the target quadrant. Each mouse was given 4 daily trials with a 20-min intertrial interval for 4 consecutive days to find the platform, and individually placed in the pool at one of 4 quadrant locations. Mice that failed to locate the escape platform within 60 s were manually guided to the platform, and allowed to stay for 20 s. The escape latency from four sessions on the same day was averaged. On the fifth day, the platform was removed to allow for probe testing. During the 60-s session, the number of crossings over the target quadrant and the total time spent in target quadrant were recorded. Training and probe tests were analyzed using motion detection software (Actimetrics Software, Evanston, IL, USA).

### Fear conditioning test

The fear conditioning test was conducted on the next day after the Morris water maze experiments were completed, using a standard fear conditioning chamber with metal grid floor [28]. Mice were allowed to the chamber for 2 min prior to 30-s mono-frequency sound (2 kHZ, 80 dB); during the last 2 s of the sound, foot shock (1 mA) was delivered. After 3 min, this process was repeated once. On the next day, mice were placed in the same chamber again, but without any stimulation. Freezing was defined as a completely immobile posture except for respiration [29].

### Transmission electron microscopy

Transmission electron microscopy was performed as described previously [30]. Hippocampal tissue (1 mm^3^) was fixed with 2.5% glutaraldehyde at 4 ℃ for 12 h, washed in 0.1 M cacodylate buffer three times, post-fixed in 1% osmium tetroxide for 2 h, and then dehydrated through a graded series of acetone solutions (30%, 50%, 70%, 80%, 90% and 100%). Tissues were cut into 50-nm sections using an ultrathin microtome (Leica, Wetzlar, Germany), contrasted with 2% uranyl acetate for 10 min and lead citrate for 5 min, and observed under an FEI Tecnai G2 Spirit Bio TWIN transmission electron microscope (Thermo Fisher Scientific).

### Western blotting

Tissue or cells were lysed in a lysis buffer (Thermo Fisher Scientific), separated with 10% SDS-PAGE, and then transferred to polyvinylidene fluoride membranes (Thermo Fisher Scientific). Membranes were blocked 2% bovine serum albumin (BSA; Thermo Fisher Scientific) for 2 h, then incubated overnight at 4 ℃ with a primary antibody against CXCR5 (1:2000), beclin-1 (1:1000), Atg-5 (1:2000), p62 (1:2000), Iba-1 (1:1000), CD86 (1:1000), CD206 (1:1000), IL-1β (1:500), IL-6 (1:500) or GAPDH (1:1000) (all from Abcam, Cambridge, MA, UK); or against LC3 (1:1000), p38MAPK (1:1000), p-p38MAPK (Thr180) (1:1000), NF-κB p65 (1:1000), p-NF-κB p65 (Ser536) (1:1000), STAT3 (1:1000) or p-STAT3 (1:2000) (all from Cell Signaling Technology, Boston, MA, USA). After thorough washing, blots were incubated with an appropriate peroxidase-labeled secondary antibody (1:500; Abcam) for 1 h at room temperature. Protein bands were detected using enhanced chemiluminescence (Bio-Rad Laboratories, Hercules, CA, USA) and quantitated using Image J software (NIH, Bethesda, MD, USA). Band intensities were normalized to GAPDH.

### Immunofluorescence

Brains were post-fixed in 4% paraformaldehyde overnight at room temperature, embedded in an optimal cutting temperature compound (Sakura Finetek, Torrance, CA, USA), and cut into 5-μm coronal sections. The sections were blocked with 1% BSA (Thermo Fisher Scientific). For LC3 or Iba-1 staining, sections were incubated overnight at 4 ℃ with a primary antibody (Abcam) against LC3 (1:200) or Iba-1 (1:100), and then with a goat anti-rabbit IgG H&L (Cy3®) (1:100; Abcam). For co-labeling of CXCR5 and Iba-1, sections were incubated overnight at 4 °C with primary antibodies (Invitrogen) against CXCR5 (1:300) and Iba-1 (1:300), then incubated with an anti-mouse or anti-rabbit IgG (1:1000; Cell Signaling Technology) conjugated with Alexa Fluor® 488 and Alexa Fluor® 594. Sections were counterstained with 4′,6-diamidino-2-phenylindole (DAPI) for 10 min at room temperature, then observed and imaged under an Axio Observer A1/D1/Z1 fluorescence microscope (Carl Zeiss, Oberkochen, Baden-Wurttemberg, Germany).

Cells on coverslips were fixed in 4% paraformaldehyde for 15 min, permeabilized with 0.5% Triton X-100 for 15 min and blocked with 1% BSA for 2 h at 37 ℃. Cells were treated overnight at 4 ℃ with primary antibodies (Abcam) against LC3 (1:200) and Iba-1 (1:500), followed by goat anti-rabbit IgG H&L (Cy3®) (1:100; Abcam) for 2 h. Nuclei were stained with DAPI. Images were taken using a TCS SP8 fluorescence microscope (Leica, Weztlar, Germany).

### Statistical analysis

Statistical analysis was performed using GraphPad Prism 9.0.0 (Graph Pad Software, San Diego, CA, USA). All continuous variables followed normal distribution (Shapiro–Wilk test; data not shown), and were reported as mean ± standard deviation (SD). Data from Morris water maze training were analyzed using two-way ANOVA for repeated-measures, followed by the Bonferroni post hoc test for multiple comparisons. All other variables were analyzed using Student's *t* test (for comparisons between two groups), or one-way ANOVA followed by Tukey’s multiple test (for comparisons among at least three groups). *P* < 0.05 was considered statistically significant.

## Results

### Sepsis induces cognitive deficits and up-regulates CXCR5

**Fig. 2:**
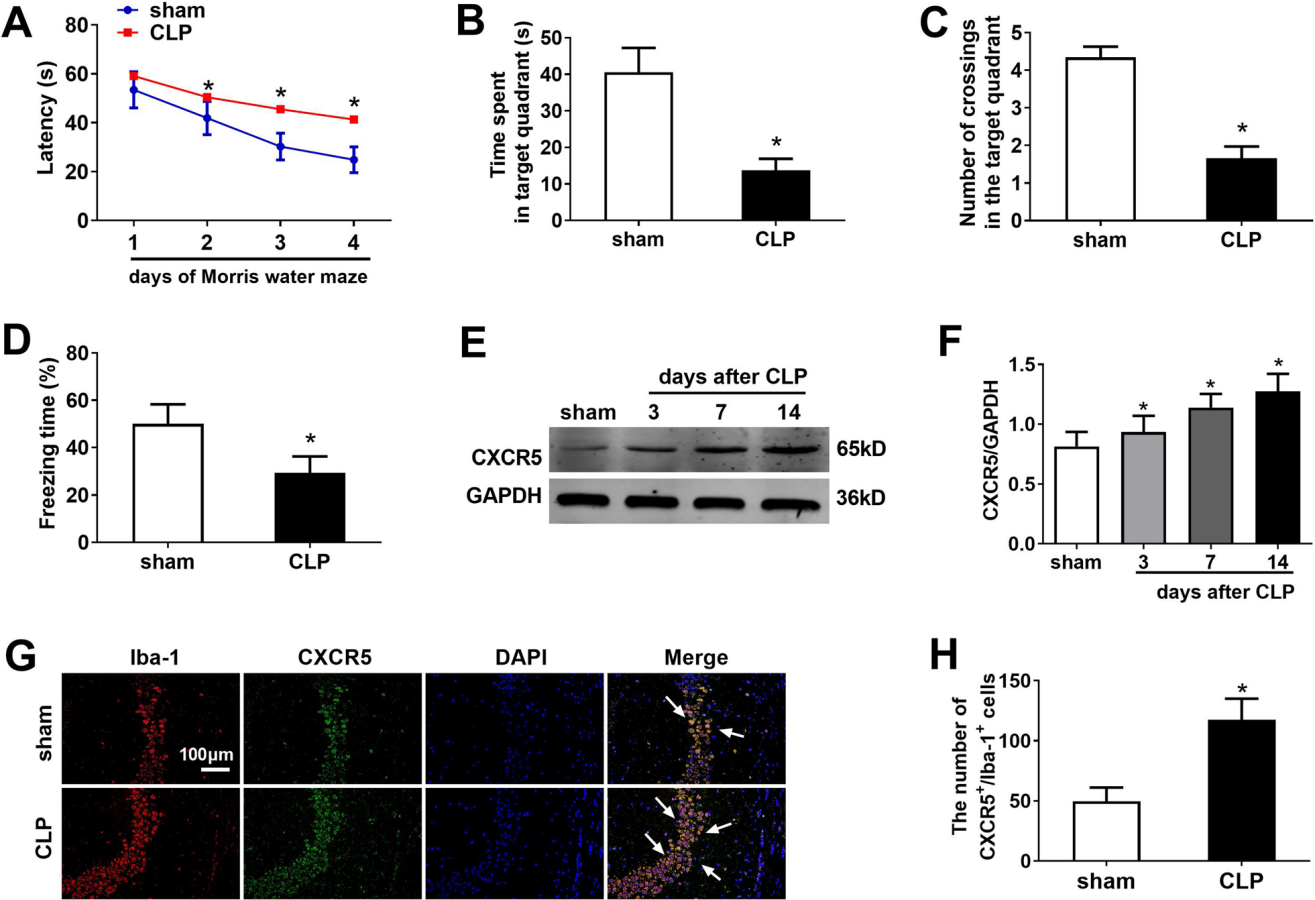
Cognitive deficits and up-regulation of CXCR5 in a mouse model of sepsis-associated encephalopathy. Adult male C57BL/6 J mice were subjected to sham surgery or CLP. **a** Escape latency time, **b** time spent in the target quadrant, and **c** number of crossings over the target quadrant in the Morris water maze beginning on day 14 after CLP. **d** Freezing time in the fear conditioning test on day 19 after CLP (*n* = 16 per group). **e, f** Western blot and densitometry of hippocampal CXCR5 on days 3, 7 and 14 after CLP (*n* = 4 per group). GAPDH was used as an internal control. **g** Double staining of CXCR5 with microglia marker Iba-1 in the hippocampus at 14 days after CLP. Arrows indicate double-stained cells. Magnification, 200 × . Scale bar, 100 μm. **h** Quantification of immunofluorescent cells co-staining positive for CXCR5 and Iba-1. *p < 0.05 vs. sham. *CLP* cecal ligation and puncture, *CXCR5* C-X-C chemokine receptor type 5, *GAPDH* glyceraldehyde-3-phosphate dehydrogenase, *Iba-1* ionized calcium binding adaptor molecule-1

In the Morris water maze, escape latency progressively decreased over the first four days of training in sham and CLP groups (Fig. 2a). The CLP group showed longer escape latencies than the sham group. The CLP group spent shorter time and had fewer crossings in the target quadrant on the test day than the sham control (Fig. 2b, c). CLP mice exhibited shorter freezing time than the sham control (Fig. 2d).

CLP mice showed progressively higher CXCR5 level in the hippocampus than sham mice on days 3, 7 and 14 after CLP surgery (Fig. 2e, f). To detect the microglial distribution of CXCR5, we co-stained for CXCR5 and Iba-1, a marker of microglia (Fig. 2g). CXCR5 colocalized with Iba-1, and CLP mouse exhibited more CXCR5^+^/Iba-1^+^ cells (Fig. 2h).

### Knocking out CXCR5 ameliorates cognitive dysfunction in SAE mice

**Fig. 3:**
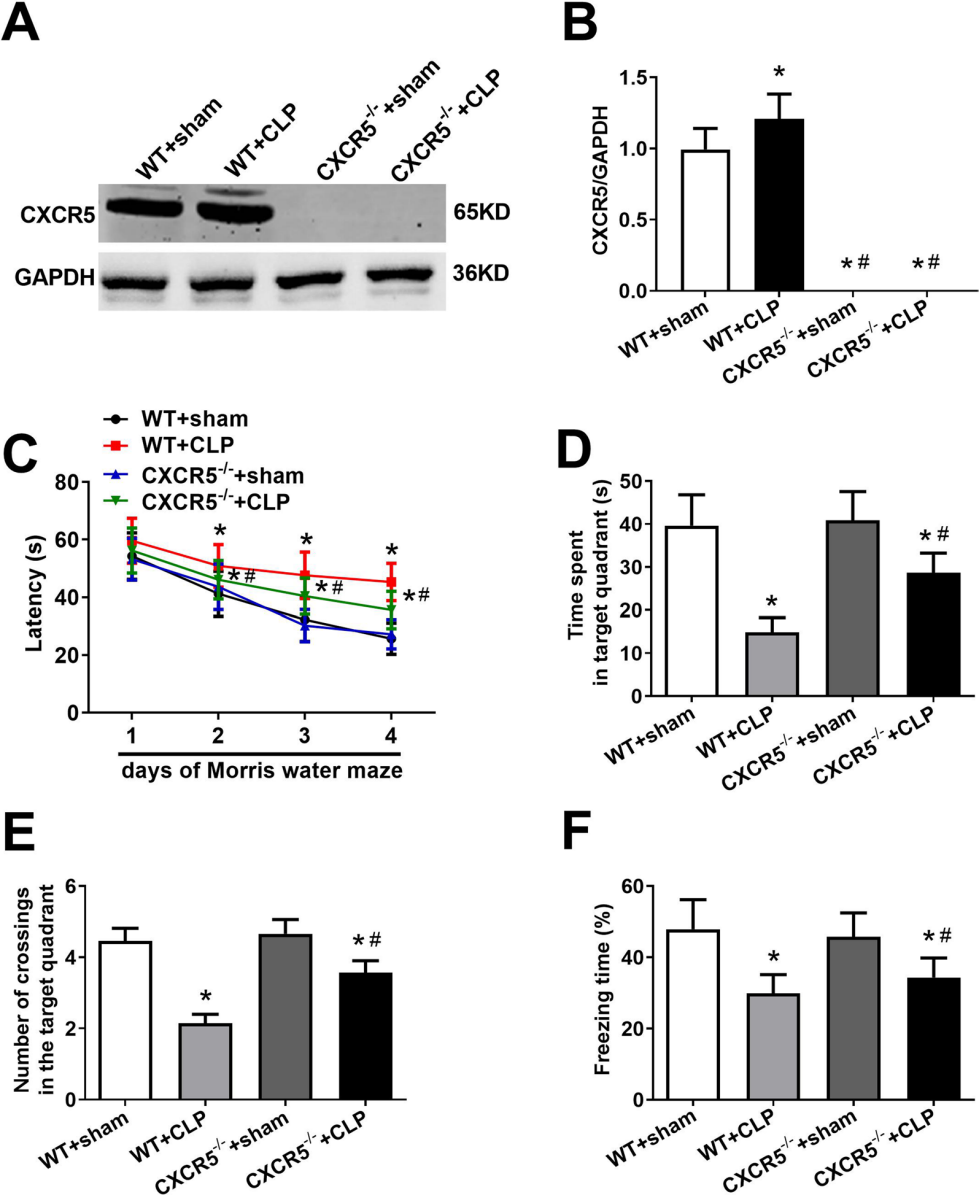
CXCR5 knockout ameliorated sepsis-induced cognitive dysfunction in mice with sepsis-associated encephalopathy. WT mice or mice lacking the CXCR5 gene (CXCR5^−/−^) were subjected to sham surgery or CLP. **a** Western blot and **b** densitometry of hippocampal CXCR5 on day 14 after CLP. GAPDH was used as an internal control (*n* = 4 per group). Mice were assessed in the Morris water maze on day 14 after CLP, followed by the fear conditioning test. **c** Escape latency time, **d** time spent in the target quadrant, and **e** number of crossings over the target quadrant in the Morris water maze. **f** Freezing time in the fear conditioning test (*n* = 16 per group). *p < 0.05 vs. WT + sham; ^#^*p* < 0.05 vs. WT + CLP. *CLP* cecal ligation and puncture, *CXCR5* C-X-C chemokine receptor type 5, *GAPDH* glyceraldehyde-3-phosphate dehydrogenase, *WT* wild-type

CLP mice showed higher hippocampal CXCR5 expression than the sham control (Fig. 3a, b), as well as longer escape latencies during Morris water maze training (Fig. 3c). Escape latencies were shorter in *CXCR5*^*−/−*^ mice that underwent CLP than in WT mice with CLP. CLP mice spent shorter time and had fewer crossings in the target quadrant on the test day than the sham control, whereas *CXCR5*^*−/−*^ mice that underwent CLP spent longer time in the target quadrant and made more crossings than CLP mice (Fig. 3d, e). CLP mice showed shorter freezing time than the sham control, whereas *CXCR5*^*−/−*^ mice that underwent CLP showed longer freezing time than CLP mice (Fig. 3f).

### Knocking out CXCR5 promotes hippocampal autophagy in SAE mice

**Fig. 4:**
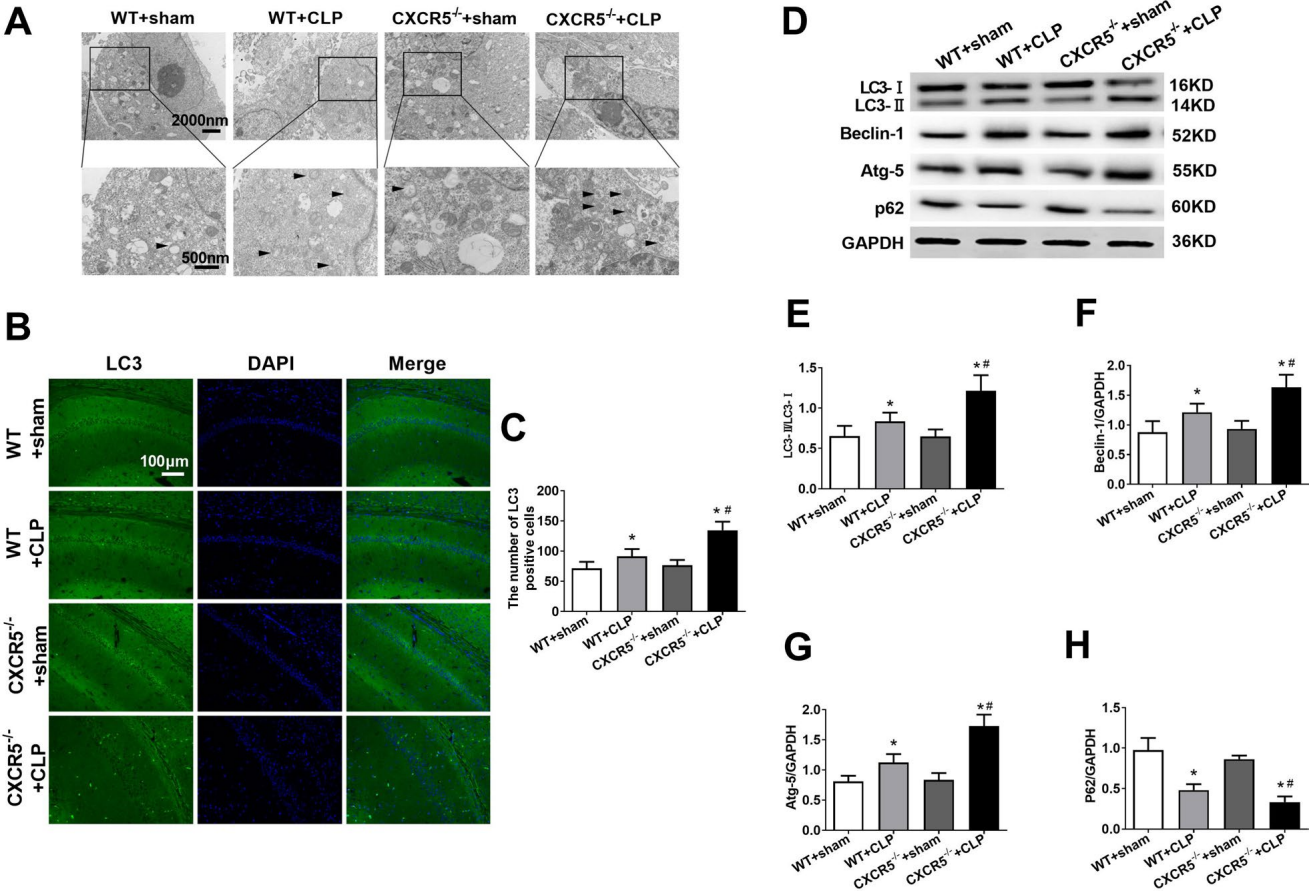
CXCR5 knockout restored hippocampal autophagy in mice with sepsis-associated encephalopathy. WT mice or mice lacking the CXCR5 gene (CXCR5^−/−^) were subjected to sham surgery or CLP. **a** Representative transmission electron micrographs of the hippocampus show zoomed-in views (magnification, 5000 × ; Scale bar, 500 nm) of full image of a cell (magnification, 2000 × ; Scale bar, 2000 nm). Arrows indicate autophagosome. **b** Representative immunofluorescence micrographs showing LC3 in the hippocampal CA1 region. Magnification, 200 × . Scale bar, 100 μm. **c** Quantification of immunofluorescent cells staining positive for LC3. **d**–**h** Western blot and densitometry of hippocampal LC3, beclin-1, Atg-5 and p62, and the ratio of LC3-II/LC3-I. GAPDH was used as an internal control (*n* = 4 per group). *p < 0.05 vs. WT + sham; ^#^*p* < 0.05 vs. WT + CLP. *Atg-5* autophagy-related gene-5, *CLP* cecal ligation and puncture, *CXCR5* C-X-C chemokine receptor type 5, *GAPDH* glyceraldehyde-3-phosphate dehydrogenase, *LC3* microtubule-associated protein 1 light chain 3, *WT* wild-type

Transmission electron microscopy demonstrated more autophagosomes in the hippocampus from CLP mice than the sham control (Fig. 4a). The number was even higher in *CXCR5*^*−/−*^ mice that underwent CLP. Similarly, the hippocampus of CLP mice had higher numbers of LC3-positive cells and a higher LC3-II/LC3-I ratio than the sham control; these parameters were even higher in *CXCR5*^*−/−*^ mice that underwent CLP (Fig. 4b-e). The number of LC3-expressing cells or the LC3-II/LC3-I ratio did not differ between WT and *CXCR5*^*−/−*^ mice that underwent sham surgery.

Regardless of the genotype, mice that underwent CLP showed significantly higher levels of beclin-1 and Atg-5 and lower levels of p62 in the hippocampus than the sham control (Fig. 4d, f–h). These CLP-induced changes were even greater in *CXCR5*^*−/−*^ mice.

### Knocking out CXCR5 attenuates sepsis-induced microglial polarization in hippocampus

**Fig. 5:**
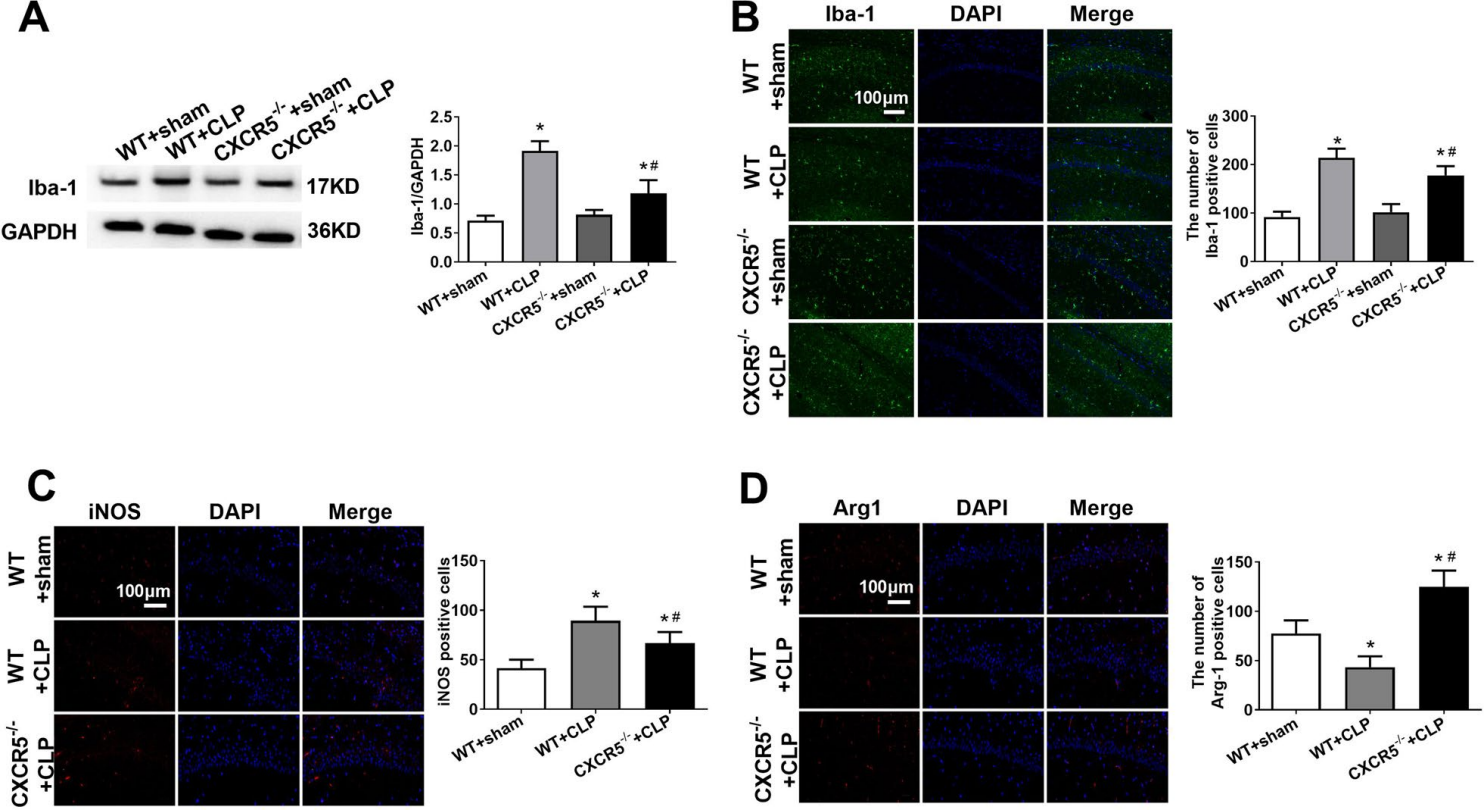
CXCR5 knockout reversed the alteration in hippocampal microglial M1/M2 polarization in mice with sepsis-associated encephalopathy. WT mice or mice lacking the CXCR5 gene (CXCR5^−/−^) were subjected to sham surgery or CLP. **a** Western blot and densitometry of hippocampal Iba-1 on day 14 after CLP. **b** Representative micrographs of immunohistochemistry for Iba-1 in the hippocampal CA1 and the number of Iba-1-positive cells. Magnification, 200 × . Scale bar, 100 μm. Representative immunofluorescence micrographs and quantitation (*n* = 4 per group) after staining for **c** M1 marker iNOS and **d** M2 marker Arg-1 in the hippocampal CA1 region. Magnification, 200 × . Scale bar, 100 μm. *p < 0.05 vs. WT + sham; ^#^*p* < 0.05 vs. WT + CLP. *Arg-1* arginase-1, *CLP* cecal ligation and puncture, *CXCR5* C-X-C chemokine receptor type 5, *Iba-1* ionized calcium binding adaptor molecule-1, *iNOS* inducible nitric oxide synthase, *WT* wild-type

In WT mice, CLP increased both Iba-1 and Iba-1-positive cells (Fig. 5a). Such increases were less pronounced in *CXCR5*^*−/−*^ mice (Fig. 5b). CLP also increased the M1 marker iNOS and decreased the M2 marker Arg-1 in WT mice, but less so in CXCR5 knockout mice (Fig. 5c, d).

### Knocking out CXCR5 attenuates sepsis-induced up-regulation of phosphorylated p38MAPK, IL-1β and IL-6 in hippocampus

**Fig. 6:**
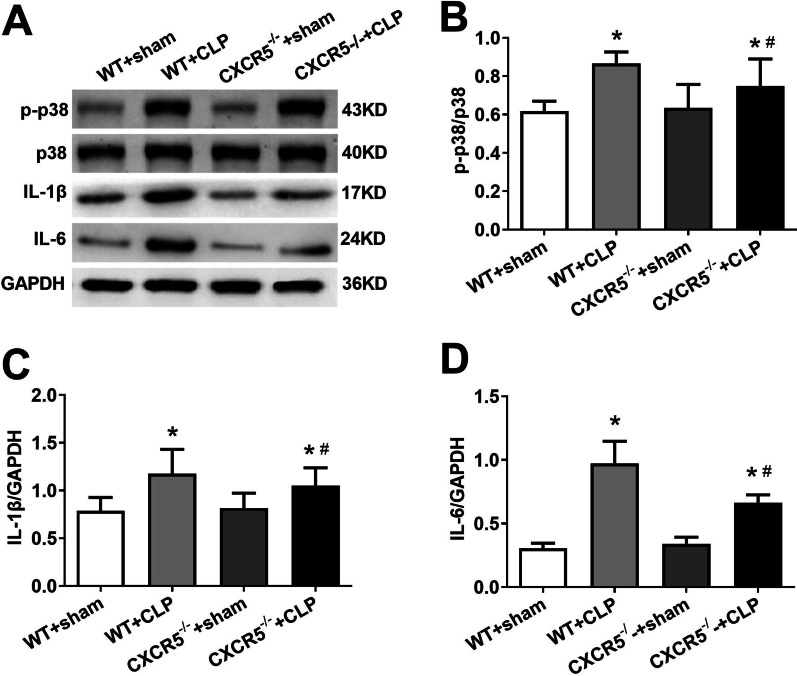
CXCR5 knockout decreased hippocampal p-p38MAPK, IL-1β and IL-6 levels in mice with sepsis-associated encephalopathy. WT mice or mice lacking the CXCR5 gene (CXCR5^−/−^) were subjected to sham surgery or CLP. **a**–**d** Western blot and densitometry of hippocampal p-p38MAPK, p38MAPK, IL-1β and IL-6 (*n* = 4 per group). *p < 0.05 vs. WT + sham; ^#^*p* < 0.05 vs. WT + CLP. *CLP* cecal ligation and puncture, *CXCR5* C-X-C chemokine receptor type 5, *IL-1β* interleukin (IL)-1β, *IL-6* interleukin (IL)-6, *p38MAPK* mitogen-activated protein kinase, *p-p38MAPK* phosphorylated p38MAPK, *WT* wild-type

CLP increased the levels of phosphorylated p38MAPK, IL-1β and IL-6 in the hippocampus in WT mice; the effects were less pronounced in *CXCR5*^*−/−*^ mice (Fig. 6).

### Knocking down CXCR5 activates p38MAPK-dependent autophagy in microglial cultures treated with LPS

**Fig. 7:**
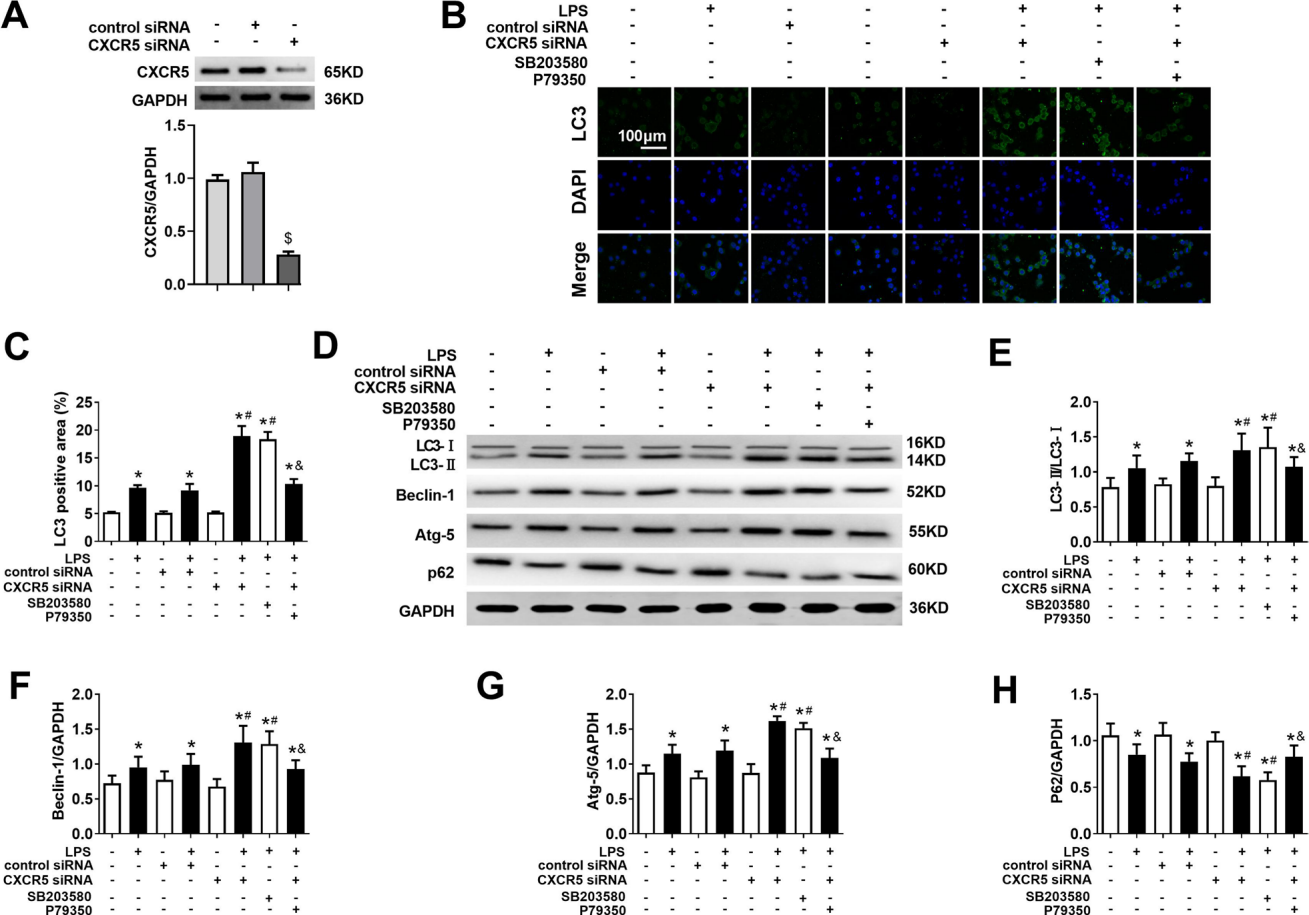
CXCR5 knockdown restored p38MAPK-dependent autophagy in LPS-treated microglial cultures. Microglial cells were treated with CXCR5 siRNA. **a** Western blot and densitometry of CXCR5 in microglia at 24 h after CXCR5 siRNA administration. ^$^*p* < 0.05 vs. cells treated without siRNAs. Microglial cells were treated with CXCR5 siRNA at 24 h before LPS treatment. Some cultures were also treated with p38MAPK inhibitor SB203580 or agonist P79350 at 1 h before siRNA administration. **b** Representative micrographs of immunohistochemistry for LC3 in microglia at 24 h after LPS treatment. Magnification, 200 × . Scale bar, 100 μm. **c** Calculation of LC3-positive area. **d**–**h** Western blot and densitometry of LC3, beclin-1, Atg-5 and p62, and the ratio LC3-II/LC3-I (*n* = 4 per group). *p < 0.05 vs. control; ^#^*p* < 0.05 vs. LPS; ^&^*P* < 0.05 vs. LPS + CXCR5 siRNA. *Atg-5* autophagy-related gene-5, *GAPDH* glyceraldehyde-3-phosphate dehydrogenase, *LC3* microtubule-associated protein 1 light chain 3

The transfection and working efficiency of CXCR5 siRNA is shown as Fig. 7a. LPS challenge triggered a significant increase in levels of LC3, beclin-1 and Atg-5; an increase in the LC3-II/LC3-I ratio; and a decrease in p62 in primary microglial cultures (Fig. 7b–h). Such effects were augmented by either CXCR5 knockdown or treatment with the p38MAPK inhibitor SB203580. Conversely, the p38MAPK agonist P79350 mitigated the effects of CXCR5 knockdown on autophagy.

### Knocking down CXCR5 attenuates LPS-induced microglial polarization and inflammatory cytokine production in microglial cultures

**Fig. 8:**
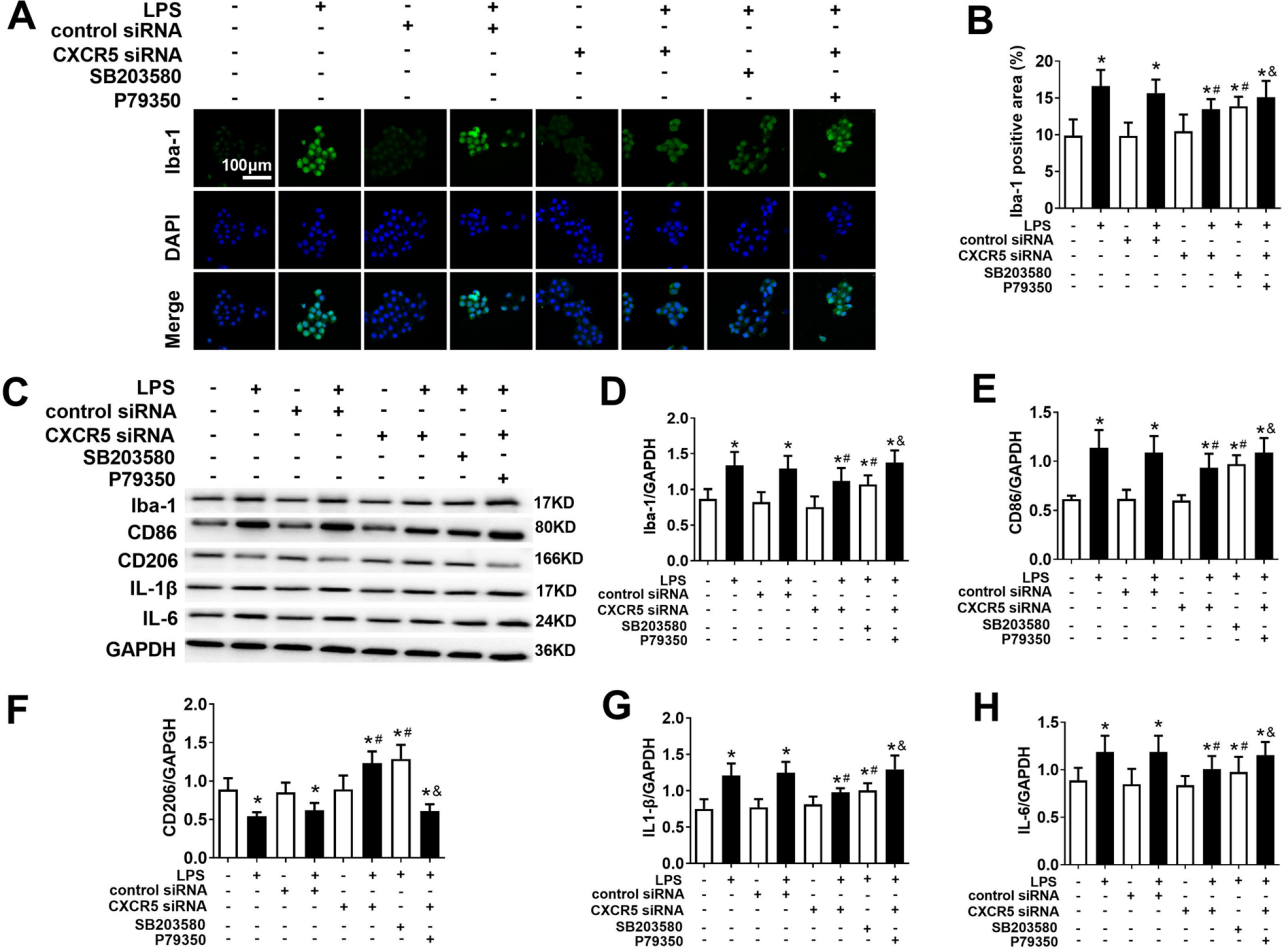
CXCR5 knockdown reversed the alteration of p38MAPK-dependent microglial M1/M2 polarization and production of inflammatory cytokines in LPS-treated microglial cultures. **a** Representative micrographs of immunohistochemistry for Iba-1 in the microglia. Magnification, 200 × . Scale bar, 100 μm. **b** Calculation of Iba-1-positive area. **c**–**h** Western blot and densitometry of Iba-1, CD86, CD206, IL-1β and IL-6 (*n* = 4 per group). *p < 0.05 vs. control; ^#^*p* < 0.05 vs. LPS; ^&^*P* < 0.05 vs. LPS + CXCR5 siRNA. *GAPDH* glyceraldehyde-3-phosphate dehydrogenase, *Iba-1* ionized calcium binding adaptor molecule-1, *IL-1β* interleukin-1β, *IL-6* interleukin-6, *LPS* lipopolysaccharide

LPS triggered an increase in Iba-1, an increase in the M1 marker CD86 and a decrease in the M2 marker CD206 (Fig. 8a–f). CXCR5 knockdown or the p38MAPK inhibitor SB203580 mitigated these changes. Conversely, the p38MAPK agonist P79350 mitigated the effects of CXCR5 knockdown on microglial polarization.

LPS triggered increases in the levels of the inflammatory cytokines IL-1β and IL-6 (Fig. 8c). Such effects were attenuated by CXCR5 knockdown or the p38MAPK inhibitor SB203580 (Fig. 8g, h). Conversely, the p38MAPK agonist P79350 mitigated the effects of CXCR5 knockdown on inflammatory cytokine production.

### Knocking down CXCR5 attenuates LPS-induced p38MAPK/NF-κB/STAT3 signaling in microglial culture

**Fig. 9:**
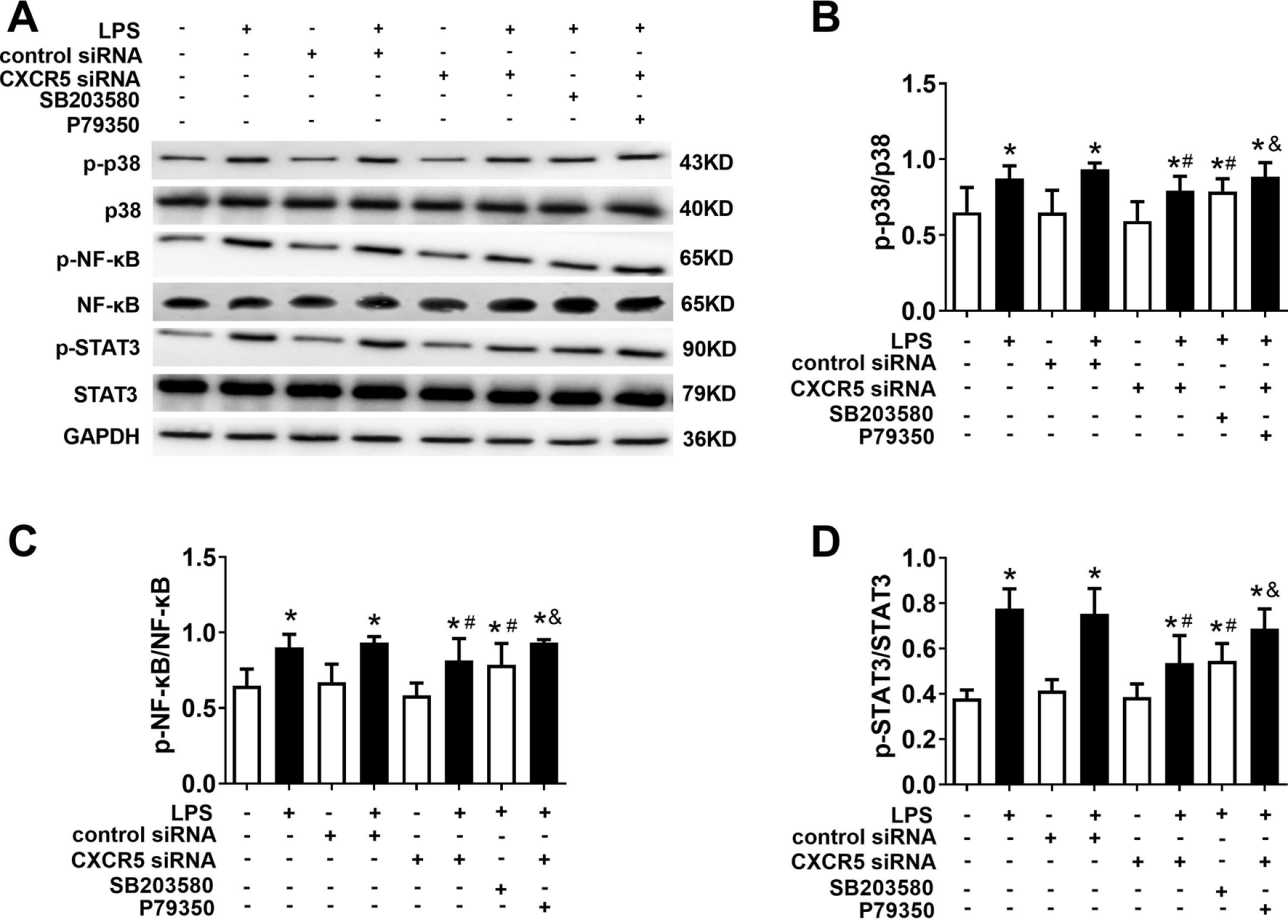
CXCR5 knockdown inhibited activation of p38MAPK-dependent NF-κB/STAT3 signaling in LPS-treated microglial cultures. **a**–**d** Western blot and densitometry of p-p38MAPK, p38MAPK, p-NF-κB, NF-κB, p-STAT3 and STAT3 and relevant ratios (*n* = 4 per group). *p < 0.05 vs. control; ^#^*p* < 0.05 vs. LPS; ^&^*P* < 0.05 vs. LPS + CXCR5 siRNA. *GAPDH* glyceraldehyde-3-phosphate dehydrogenase, *LPS* lipopolysaccharide, *NF-κB* nuclear factor kappa-B, *p38MAPK* p38 mitogen-activated protein kinase, *p-* phosphorylated, *STAT3* signal transducer and activator of transcription

LPS triggered increases in the levels of phosphorylated p38MAPK, NF-κB and STAT3; the effects were attenuated by either CXCR5 knockdown or the p38MAPK inhibitor SB203580 (Fig. 9). Conversely, the p38MAPK agonist P79350 mitigated the effects of CXCR5 knockdown.

### Autophagy inhibition attenuated the effects of CXCR5 knockdown on microglial polarization and inflammatory cytokines production in LPS-treated microglial cultures

**Fig. 10:**
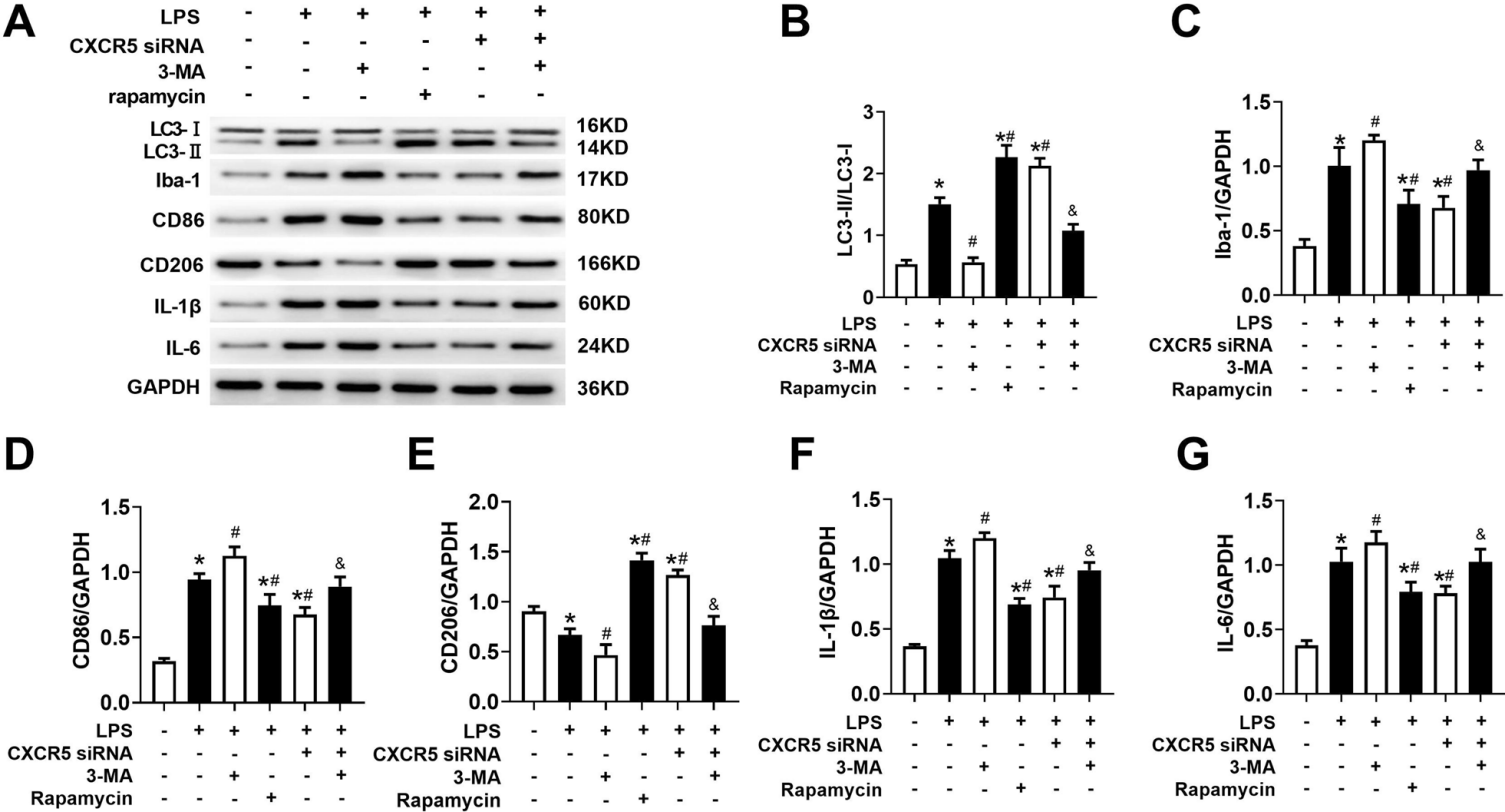
Autophagy inhibition attenuated the effects of CXCR5 knockdown on microglial polarization and inflammatory cytokines production in LPS-treated microglial cultures. **a**–**g** Western blot and densitometry of LC3, Iba-1, CD86, and CD206, IL-1β, IL-6 and the ratio of LC3-II/LC3-I (*n* = 4 per group). *p < 0.05 vs. control; ^#^*p* < 0.05 vs. LPS; ^&^*P* < 0.05 vs. LPS + CXCR5 siRNA. *CXCR5* C-X-C chemokine receptor type 5, *GAPDH* glyceraldehyde-3-phosphate dehydrogenase, *Iba-1* ionized calcium binding adaptor molecule-1, *IL-1β* interleukin (IL)-1β, *IL-6* interleukin (IL)-6, *LC3* microtubule-associated protein 1 light chain 3, *LPS* lipopolysaccharide, *3-MA* 3-methyladenine

LPS triggered an increase in the LC3-II/LC3-I ratio, an increase in level of Iba-1, an increase in the M1 marker CD86 and a decrease in the M2 marker CD206 (Fig. 10a–e). CXCR5 knockdown or the autophagy agonist rapamycin mitigated these changes. Conversely, the autophagy inhibitor 3-MA mitigated the effects of CXCR5 knockdown on microglial polarization.

LPS triggered increases in the levels of the inflammatory cytokines IL-1β and IL-6 (Fig. 10a). Such effects were attenuated by CXCR5 knockdown or the autophagy agonist rapamycin (Fig. 10f, g). Conversely, the autophagy inhibitor 3-MA mitigated the effects of CXCR5 knockdown on inflammatory cytokine production.

## Discussion

Results from the present study suggest that CXCR5 contributes to cognitive impairment by enhancing p38MAPK/NF-κB/STAT3 signaling. We further showed that CXCR5 knockout could restore autophagy, promote the microglial polarization to the M2 phenotype, and inhibit both proinflammatory cytokine release and p38MAPK activation in the hippocampus of the SAE mice. These effects were associated with attenuated cognitive dysfunction after CLP. In primary microglial cultures, we showed that CXCR5 knockdown could partially reverse LPS-induced phosphorylation of p38MAPK as well as p38MAPK-dependent up-regulation of NF-κB and STAT3, ultimately restoring autophagy activation and inhibiting proinflammatory cytokine production.

CLP resulted in prolonged latency as well as decreased time and fewer crossings in the target quadrant in the Morris maze test, and decreased freezing time in the conditioned fear test. In addition, sepsis induced microglial M1 polarization and production of proinflammatory cytokines in mouse hippocampus, as well as in primary microglia cultures. These findings are consistent with our previous work [28]. Growing evidence indicates that microglia are rapidly activated in response to septic challenge, and these cells produce substantial amounts of inflammatory cytokines [10]. Microglia-mediated neuroinflammation has a major role in the development of long-term cognitive dysfunction after sepsis [31]. Sepsis patients with encephalopathy have higher IL-6 levels in cerebrospinal fluid than non-septic controls without encephalopathy [32]. Patients who die during septic shock show strong, localized up-regulation of the microglial M1 polarization marker CD68 in the hippocampus [33]. Sepsis also up-regulates the inflammatory cytokines TNF-α, IL-6 and HMGB1 in BV-2 microglia cultures and animals [10]. The present experiments in vivo and in vitro showed that sepsis induced the activation of microglial M1 polarization and the production of pro-inflammatory cytokines. These results provide evidence for a critical role of microglia-mediated neuroinflammation in SAE. Promoting the conversion of microglia M1 polarization to the M2 phenotype [13] or inhibiting neuroinflammation [11, 28] can alleviate cognitive deficits and functional decline in SAE animals.

Autophagy is activated by the systemic inflammatory response in the septic hippocampus [19]. When autophagy is activated, LC3-I in the cytoplasm is converted to LC3-II, which aggregates on the autophagosome membrane; p62/SQSTM1 is the adaptor protein linking ubiquitin, LC3 and the autophagosome, and these proteins form a complex, which is degraded by lysosomes as a substrate of autophagy [34]. In this study, we found that sepsis induced more double-membrane autophagosomes, the shift from LC3-I to LC3-II, increased levels of beclin-1 and Atg-5, and reduction in free p62 in the mouse hippocampus, indicating the activation of hippocampal autophagy. Clinical and preclinical studies suggest that a certain degree of autophagy can protect the body from sepsis-induced injury. For example, pyrrolidine dithiocarbamate can increase autophagy in the hippocampus, helping protect brain tissue from sepsis-induced injury in rats [19]. Suppression of autophagy has been linked to worse clinical outcomes in patients with severe sepsis [35]. Impairment in autophagosome–lysosome fusion, which stalls autophagy, may contribute to sepsis-induced brain injury [36]. This literature and our findings suggest that increasing levels of autophagy may help mitigate sepsis-induced brain injury. However, a balance is needed: excessive autophagy can destroy cell homeostasis [37]. Further work should explore what autophagy levels may exert the best therapeutic effects. In any case, our findings confirm that autophagy is deregulated in the hippocampus of sepsis animals.

The same upstream signals may trigger autophagy and subsequent microglia-mediated neuroinflammation in sepsis. CXCR5, the only known receptor for the chemokine CXCL13, is known to mediate neuroinflammation and thereby contribute to impairment of learning and memory in intractable temporal lobe epilepsy patients and pilocarpine-induced epileptic rats [38]. The CXCL13/CXCR5 signaling axis contributes to neurodegeneration in individuals with cognitive deficit disease [38], as well as in animal models with neurodegeneration [23], as we have confirmed in our previous work [22, 39]. Infection of the CNS induces production of CXCL13 in microglia, macrophages, and endothelial cells there [40, 41]. CXCR5 deficiency in retinal pigment epithelial cells has been shown to deregulate autophagy, as reflected in decreased LC3B-II, increased p62, abnormal autophagosomes and impaired lysosome enzymatic activity [21]. This dysregulation of autophagy contributes to age‐related macular degeneration in mice [42]. Our results suggest that CXCR5-mediated dysregulation of autophagy may also contribute to cognitive deficits in SAE. Our results further suggest that CXCR5 deficiency restores autophagy and inhibits microglia-induced neuroinflammation, ameliorating sepsis-induced cognitive dysfunction. Similarly, CXCR5 deficiency in mice reduces inflammatory pain and decreases activation of spinal microglia and astrocytes in a mouse model of neuropathic pain induced by peripheral injection of complete Freund's adjuvant [43].

**Fig. 11:**
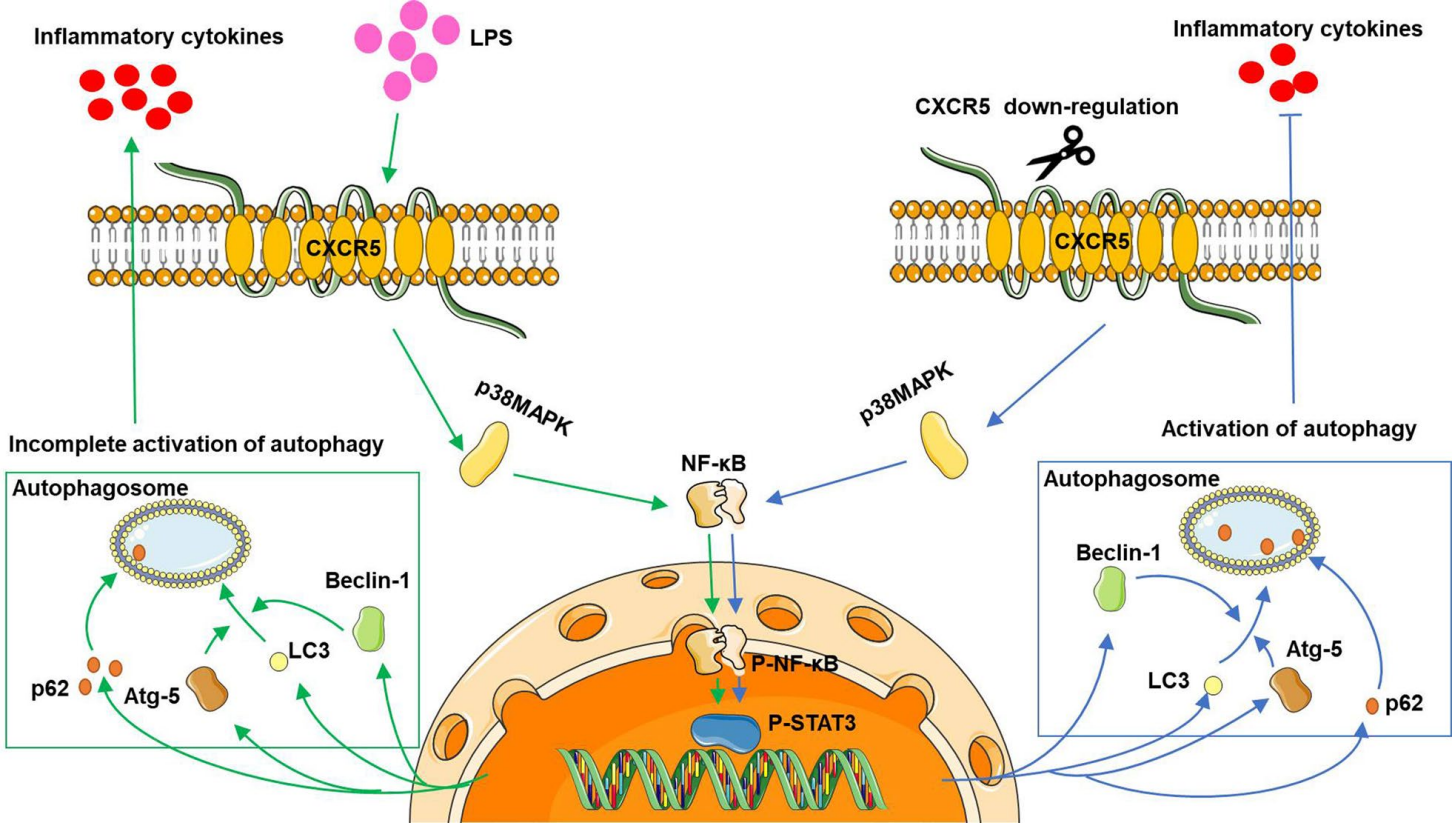
Schematic diagram depicting the possible mechanisms through which CXCR5 regulates autophagy. MAPK signaling downstream of CXCR5 induced by LPS promotes the up-regulation of NF-κB/STAT3 axis. Consequently, activation of NF-κB/STAT3 pathway contributes to incomplete activation of autophagy and production of inflammatory cytokines, thereby impairs learning and memory function. CXCR5 down-regulation results in inhibition of MAPK/NF-κB/STAT3 axis and activation of autophagy in brain, which leads to the improved cognitive function in mice with sepsis-associated encephalopathy. *Atg-5* autophagy-related gene-5, *CXCR5* C-X-C chemokine receptor type 5, *LC3* microtubule-associated protein 1 light chain 3, *LPS* lipopolysaccharide, *NF-κB* nuclear factor kappa-B, *p38MAPK* mitogen-activated protein kinase, *p-* phosphorylated, *STAT3* signal transducer and activator of transcription

We found that sepsis induced the phosphorylation of p38MAPK in the hippocampus in vivo and in microglia cultures. CXCR5 deficiency partially reversed this phosphorylation, inhibiting downstream activation of NF-κB and STAT3. Our results are consistent with other studies involving the CXCR5/CXCL13 axis. In a mouse model of neuropathic pain, CXCL13 acts via CXCR5 to activate p38MAPK signaling, triggering orofacial neuropathic pain and promoting neuroinflammation [23]. Sepsis in neonatal rats has been shown to activate p38MAPK signaling in the brain, and the p38MAPK inhibitor SB203580 can protect against sepsis-associated cognitive deficits [44]. CXCL13 level was increased in the serum of patients with sepsis, and CXCL13 also acts via p38MAPK signaling to drive LPS-induced hyperpermeability of the endothelium in human umbilical vein endothelial cells, suggesting that targeting CXCL13 may alleviate sepsis [45]. Similarly, p38MAPK mediates LPS-induced morphological changes and production of IL-1β in primary microglial cultures and the brain [24], and it contributes to acute lung injury in a mouse model by stimulating autophagy, oxidative stress and inflammatory responses [46]. Our results add SAE to the list of diseases in which p38MAPK drives pathophysiology and may therefore be a useful therapeutic target. Analogously, inhibiting p38MAPK using SB203580 has been shown in vitro to reverse the dysregulation of NF-κB and downstream STAT3 and to slow pancreatic tumor growth [47]. Therefore, as shown in Fig. 11, knocking out or down-regulating CXCR5 reduces p38MAPK activation and, consequently, its downstream signaling, ultimately ameliorating sepsis-induced cognitive defects.

There were some limitations to this study. We blocked autophagy in vitro when knockdown down CXCR5, and found that autophagy plays an important role in the protective effect of CXCR5. There is still lack of direct in vivo evidence showing that CXCR5 down-regulation alters cognitive function via autophagy. However, we did not investigate the role of autophagy in the protective effect of CXCR5 in vivo, warranting further investigation. AMPK/mTOR is the most classical pathway involved in the autophagy regulation [48], and the MAPK or NF-κB pathways should be an indirect factor regulating the autophagy pathway [23]. A recent report also suggests that the PI3K/AKT/FOXO1 signaling axis mediates autophagy inhibition by CXCR5 in retinal pigment epithelial cells. Whether CXCR5 regulated autophagy via AMPK/mTOR pathway remains unknown, warranting further investigation. Additionally, potential implication of the CXCR5/p38MAPK axis in infection-induced sepsis should be explored in future studies.

## Conclusion

Our results in a mouse model of SAE and in primary microglial cultures suggest that sepsis up-regulates hippocampal CXCR5, which contributes to incomplete activation of autophagy, polarization of microglia toward the M1 phenotype, production of inflammatory cytokines and appearance of cognitive deficits. Our results further suggest that down-regulating CXCR5 can restore autophagy, polarize microglia toward the M2 phenotype, and inhibit p38MAPK/NF-κB/STAT3 signaling, ultimately attenuating sepsis-induced neuroinflammation and cognitive dysfunction. In this way, our study provides the strongest evidence so far that targeting the CXCR5/p38MAPK axis may help treat or even prevent neurodegeneration in SAE.

## Data Availability

All data generated or analyzed during this study are available from the corresponding author upon request.

## Abbreviations

Arg-1: Arginase-1
Atg-5: Autophagy-related protein 5
CLP: Cecal ligation and perforation
CXCL13: C-X-C motif chemokine 13
CXCR5: C-X-C motif chemokine receptor 5
LC3: Microtubule-associated protein 1 light chain 3
MAPK: Mitogen-activated protein kinase
IL-1β: Interleukin-1 beta
iNOS: Inducible nitric oxide synthase
NF-κB: Nuclear factor kappa-B
RT-qPCR: Reverse transcription-quantitative polymerase chain reaction
SAE: Sepsis-associated encephalopathy
STAT3: Signal transducer and activator of transcription
TNF-α: Tumor necrosis factor alpha
WT: Wild-type
